# Chromogranin A (CgA) as Poor Prognostic Factor in Patients with Small Cell Carcinoma of the Cervix: Results of a Retrospective Study of 293 Patients

**DOI:** 10.1371/journal.pone.0033674

**Published:** 2012-04-17

**Authors:** Ling-Min Liao, Xin Zhang, Yu-Feng Ren, Xiao-Ying Sun, Na Di, Nan Zhou, Rui-Ke Pan, Shu-Hua Ma, Li-Xue Zhou

**Affiliations:** 1 Department of Gynecology and Obstetrics, Sun Yat-sen Memorial Hospital, Sun Yat-sen University, Guangzhou, Guangdong, China; 2 Department of Radiotherapy, First Affiliated Hospital, Sun Yat-sen University, Guangzhou, Guangdong, China; 3 Department of Gynecology and Obstetrics, Feng Tian Hospital, Shenyang Medical College, Shenyang, Liaoning, China; 4 Department of Radiology, First Affiliated Hospital, Medical College of Shantou University, Shantou, Guangdong, China; 5 Guangdong Key Laboratory of Medical Molecular Imaging, Shantou, Guangdong, China; Ospedale Pediatrico Bambino Gesú, Italy

## Abstract

**Background:**

Small cell carcinoma of the cervix (SCCC) is a very rare tumor. Due to its rarity and the long time period, there is a paucity of information pertaining to prognostic factors associated with survival. The objective of this study was to determine whether clinicopathologic finings or immunohistochemical presence of molecular markers predictive of clinical outcome in patients with SCCC.

**Methodology and Findings:**

We retrospectively reviewed a total of 293 patients with SCCC (47 patients from Cancer Center of Sun Yat-sen University in china, 71 patients from case report of china journal, 175 patients from case report in PubMed database). Of those 293 patients with SCCC, the median survival time is 23 months. The 3-year overall survival rates (OS) and 3-year disease-free survival rates (DFS) for all patients were 34.5% and 31.1%, respectively. Univariate and multivariate analysis showed that FIGO stage (IIb–IV VS I–IIa, Hazard Ratio (HR) = 3.08, 95% confidence interval (CI) of ratio = [2.05, 4.63], *P*<0.001), tumor mass size (≥4 cm VS <4 cm, HR = 2.37, 95% CI = [1.28, 4.36], *P* = 0.006) and chromogranin A (CgA) (Positive VS Negative, HR = 1.81, 95% CI = [1.12, 2.91], *P* = 0.015) were predictive of poor prognosis. CgA stained positive was found to be highly predictive of death in early-stage (FIGO I–IIa) patient specifically.

**Conclusions:**

Patients with SCCC have poor prognosis. FIGO stage, tumor mass size and CgA stained positive may act as a surrogate for factors prognostic of survival. CgA may serve as a useful marker in prognostic evaluation for early-stage patients with SCCC.

## Introduction

Neuroendocrine small cell cervical carcinoma is an aggressive, but rare form of cervical cancer with an incidence of less than 3% of all cervical cancers [1]–[3]. These tumors are characterized by a high incidence of early nodal and distant metastases, resulting in poorer prognosis than other subtypes of cervical cancers [4]–[6]. In previous studies, 60.0–82.0% of small cell cervical carcinomas had lymph-vascular space infiltration or pelvic lymph node metastasis at the time of diagnosis [7]–[9]. Moreover, small cell cervical carcinoma exhibits a propensity for rapid distant metastasis to sites including the lung, liver, brain, bone, pancreas, and lymph nodes, resulting in treatment failure in most cases [8]–[11].

Most patients die as a result of early metastasis via the bloodstream and recurrences usually occur within 2 years. Given the poor prognosis, it is important to identify prognostic factors responsible for survival in an effort to improve treatment strategies. However, due to its rarity and the long time period required to enroll a sufficient number of patients, most studies on SCCC are comprised of only small series and case reports, making it difficult to perform a randomized, controlled clinical trial to determine optimal therapy and draw conclusions on overall management. To determine the prognostic factors of this rare tumor, we pooled cases from our own institution with all the reported cases in the literature from PubMed database and china journal to perform a meta-analysis.

## Materials and Methods

### Ethics Statement

All the 47 patients from Cancer Center, Sun Yat-Sen University agreed to participate in the study and gave written informed consent. This study was approved by the medical ethics committee of Cancer Center of Sun Yat-Sen University and complied with the declaration of Helsinki.

**Figure 1:**
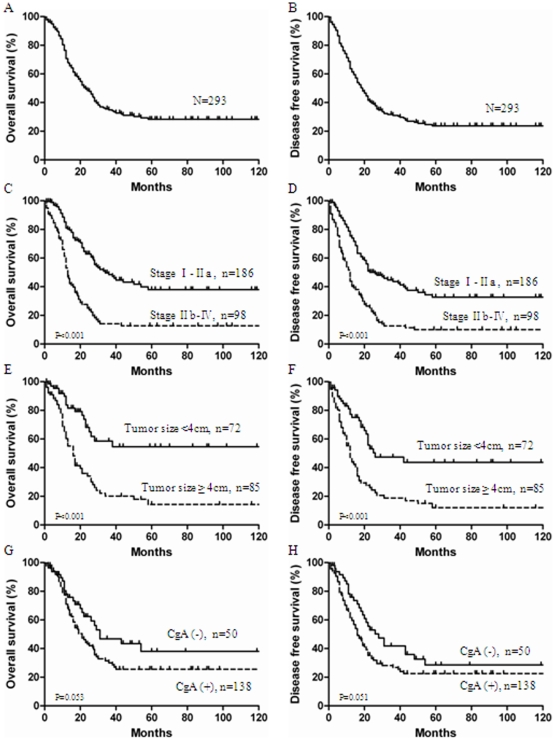
1. Relationship between clinicopathologic variables and SCCC patient survival. (**A. B**): Overall survival (OS) and disease free survival (DFS) base on all 293 patients with Small cell carcinoma of the cervix (SCCC). (**C. D**): OS and DFS based on International Federation of Gynecology and Obstetricsstage (FIGO) stage. (**E. F**): OS and DFS based on tumor size. (**G. H**): OS and DFS based on chromogranin A (CgA) express.

### Samples and Cases

In this study, a total of 293 patients with SCCC were enrolled. Forty-seven patients with SCCC who received diagnoses from 1998–2009 were identified from gynecology department of Cancer Center, Sun Yat-sen University, Guangzhou, China. The remaining 246 patients were collected from case-series reported in the literature from PubMed database and china journal. All patients were selected based on the following criteria: availability of conclusive histopathologic diagnosis as SCCC; no previous malignant disease or a second primary tumor; with complete clinical pathology and follow-up data; the immunostainings were done in the cases under the identical conditions and use the same scoring method. Cases with not good enough data or different immunohistochemistry method were excluded from our study to provide relative accurate data for meta-analysis. (**Table S1**). Patient and disease characteristics, including age of diagnosis, Federation Internationale Gynecologica Obstetrica (FIGO) stage, lymph node involvement, lymph-vascular space invasion, tumor size, depth of stromal invasion and the stained status of Neurone-specific enolase (NSE), Chromogranin A (CgA), Synaptophysin (SYN), were evaluated.

### Immunohistochemistry (IHC)

Paraffin-embedded, archived SCCC samples obtained from 40 patients (7 patients received preoperative radiotherapy and/or chemotherapy were excluded.) were histologically and clinically diagnosed from the Cancer Center, Sun Yat-Sen University. IHC staining (NSE (DAKO; 1∶500), SYN (DAKO; 1∶300), CgA (DAKO; 1∶300)) was carried out on 4-um sections. The detailed staining procedures were performed according to the reference “Zheng M et al., Obstet Gynecol 2010” [12]. Briefly, Sections were deparaffinized in xylene and rehydrated in graded ethanol and distilled water before being heated in a microwave in Tris-EDTA for 25 min. Endogenous peroxidase activity of the samples was blocked by incubating the slides in 3% hydrogen peroxide (H_2_O_2_) in methanol for 15 min. Incubation the first antibody overnight at 4°C. A biotin-conjugated goat anti-rabbit IgG secondary antibody (ZhongSan-JingQiao Biologic Technology Co., Beijing, China) was then applied for 15 min at 37°C. The last, all sections were stained in DAB for the same duration of time. Immunoreactivity was described by the percentage of positive tumor cells (percent positivity) and by the staining intensity (weak, moderate, strong). Slides given scores of (−) or (+) were recorded as negative, and slides given scores of (++) and (+++) were recorded as positive. All results were confirmed by more than 2 pathologists in a double-blind analysis. The majority of the 246 cases selected from the literature were analysed with the same or a similar immunohistochemical method (**Table S2**). For the others no indication was found.

### Statistical analysis

The association of CgA protein expression with SCCC patient's clinicopathologic features and the correlations between molecular features detected with each other were assessed by the chi-square test. Overall survival (OS) and disease-free survival (DFS) were evaluated using the Kaplan–Meier method and log-rank tests. The Cox proportional hazards model was used to estimate the independent factors prognostic for OS and DFS. All analyses were carried out using SPSS software (version 13.0, SPSS Inc., Chicago, IL). Two-sided P values of <0.05 were considered to indicate statistical significance. The end points of all 47 patients from Cancer Center, Sun Yat-sen University were updated in March 2011.

**Figure 2:**
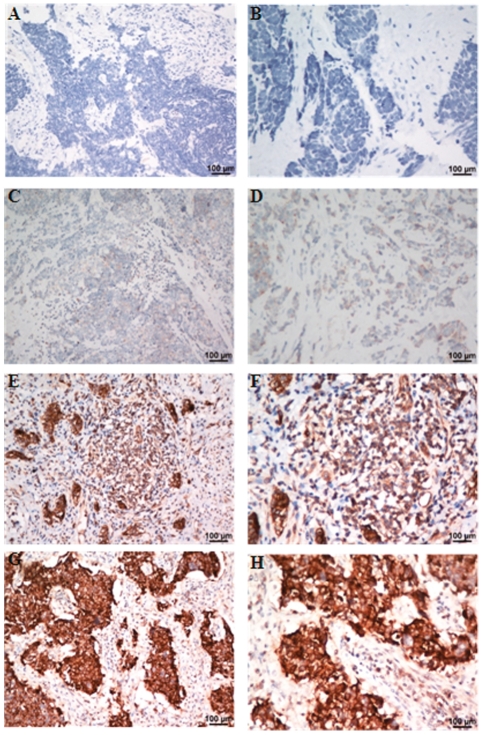
2. Immunohistochemical analyses of chromogranin A (CgA) staining in Small cell carcinoma of the cervix tissues. As can be seen, CgA shows no positive staining (−)(case-431901; **A**: x200; **B**: x400), weakly positive staining (+) (case-385737; **C**: x200; **D**: x400), moderate staining (++) (case-449570; **E**: x200; **F**: x400), strong staining (+++) (case-437412; **G**: x200; **H**: x400) in Small cell carcinoma of the cervix tissues.

## Results

### Clinicopathologic features

**Figure 3:**
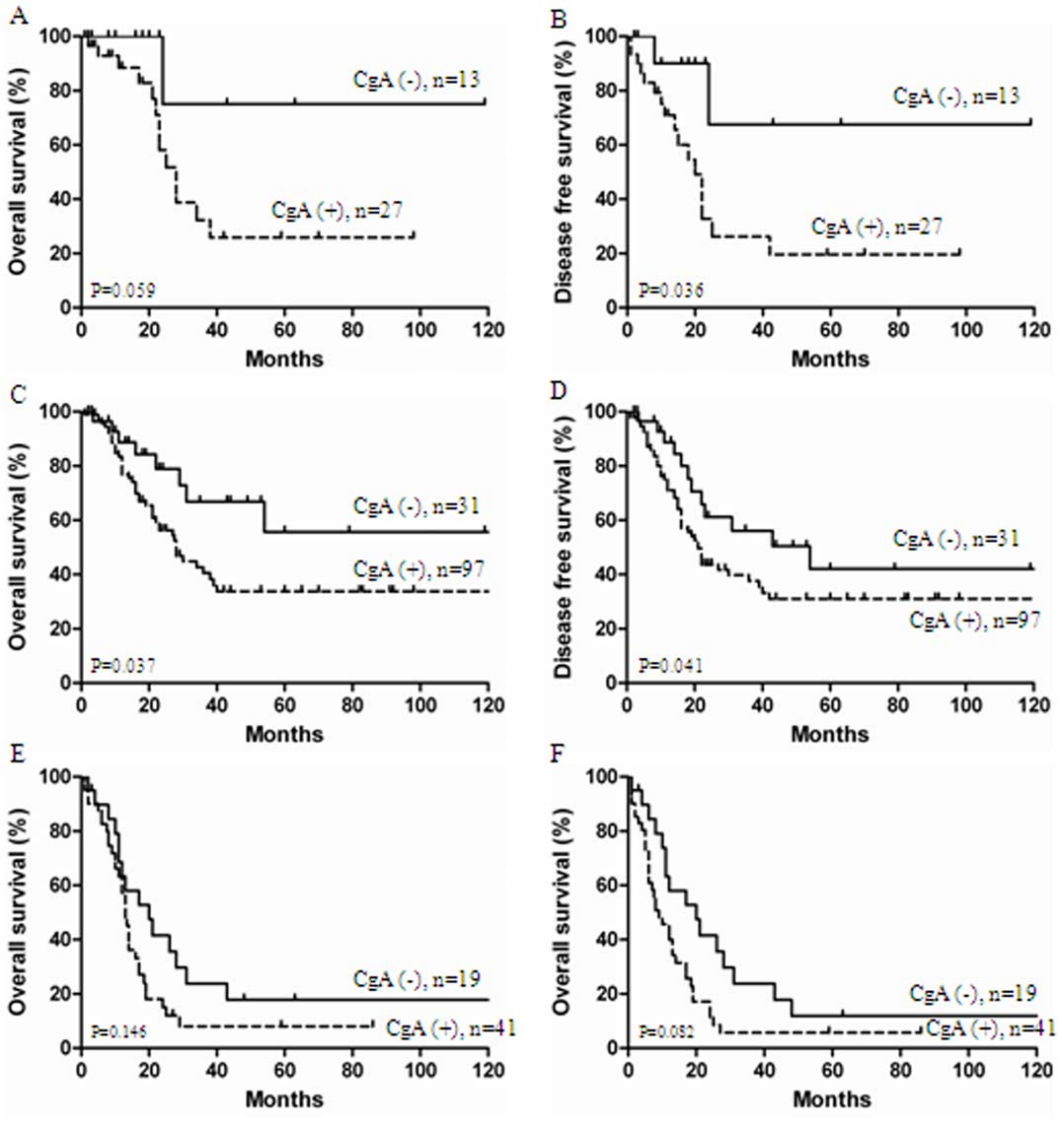
3. Survival curves stratified by chromogranin A (CgA) levels according to International Federation of Gynecology and Obstetricsstage (FIGO) stage. (**A. B**): The differences of overall survival and disease free survival curves according to CgA expression were seen in 40 patients from Cancer Center of Sun Yat-sen University in china. (**C. D**): In FIGO stage I–IIa classification panel, patients with CgA stained negative show much better overall survival and disease free survival. (**E. F**): In FIGO stage IIb–IV classification panel, patients with CgA stained negative not show much better overall survival and disease free survival.

The two hundred and ninety three patients were ranged in age from 18 to 83 years with a median of 40 years. Abnormal vaginal bleeding or vaginal discharge at presentation was noted in 75.9% of patients, 11.4% had abdominal or back pain, 2.2% had general weakness and swelling, 10.5% had no symptom. One hundred and fifty five patients (54.6%, 155/284) had FIGO stage I, 89 (31.3%, 89/284) had stage II, 24 (8.5%, 24/284) had stage III, and 16 (5.6%, 16/284) had stage IV. One hundred and thirty two (70.2%) patients had a pure histologic type composed of SCCC and 56 (29.8%) had a mixed histologic pattern associated with squamous cell carcinoma or adenocarcinoma in addition to the SCCC component. Sixty five patients (54.2%, 65/120) have lymph-vascular space infiltration and 87 (47.0%, 87/185) have lymph node metastasis in this study. However, 60.0–82.0% SCCC patients had lymph-vascular space infiltration or pelvic lymph node metastasis at the time of diagnosis in previous studies [7]–[9]. Other clinicopathologic characteristics are shown in **Table S3.**

**Table 1:** Univariate and Multivariate Cox Logistic Analysis of Factors Associated with Survival.

Clinical Variable	Subset	Hazard ratio (95% CI)	P value
**Univariate analysis (n = 293)**			
Age(years)	≥40 VS <40	1.20 (0.87–1.66)	0.277
Tumor homology	Pure VS Mixed	0.86 (0.54–1.35)	0.504
FIGO stage	IIb–IV VS I–IIa	2.86 (2.06–3.98)	**<0.001**
Tumor mass size	≥4cm VS <4cm	3.02 (1.78–5.13)	**<0.001**
Lymph node metastasis	Negative VS Positive	0.34 (0.22–0.55)	**<0.001**
Lymph-vascular space invasion	Negative VS Positive	0.69 (0.38–1.25)	0.222
Depth of stromal invasion	≥2/3 VS <2/3	2.43 (1.13–5.21)	**0.022**
Neurone-specific enolase	Positive VS Negative	1.32 (0.66–2.65)	0.432
Chromogranin A	Positive VS Negative	1.78 (1.02–2.52)	**0.034**
Synaptophysin	Positive VS Negative	1.22 (0.99–1.47)	0.057
**Multivariate analysis (n = 293)**			
FIGO stage	IIb–IV VS I–IIa	3.08 (2.05–4.63)	**<0.001**
Tumor mass size	≥4cm VS <4cm	2.37 (1.28–4.36)	**0.006**
Chromogranin A	Positive VS Negative	1.81 (1.12–2.91)	**0.015**

FIGO, International Federation of Gynecology and Obstetrics; CI, confidence interval. **Bold** indicates significant values.

### Relationship between clinicopathologic variables and SCCC patient survival

The 3-year OS rates and 3-year DFS rates for all 293 patients were 34.5% and 31.1%, respectively (**Figure 1** **. A. B**). The median survival time for all patients was 23 months (range, 1–264 months). The median survival in FIGO stage IA–IIA and IIB–IV were 34 months and 13 months, respectively (*P*<0.001) (**Figure 1** **. C. D**). Patients without lymph node metastases had a 3-year OS rates of 54.1%, compared with 23.4% for patients with lymph node metastases (*P*<0.001); Patients with tumors <4 cm had a 3-year OS rates of 58.4% vs 20.1% in those with larger tumors (*P*<0.001) (**Figure 1** **. E. F**). In contrast, age (*P* = 0.270), lymph vascular space invasion (*P* = 0.214), tumor homology (*P* = 0.499) and NSE stained positive (*P* = 0.426) were not prognostic for survival. Although not statistically significant, CgA stained positive was tended to adversely affect survival. Patients with CgA stained positive had a 3-year survival rate of 46.8%, compared with 30.0% for patients with CgA stained negative (P = 0.053) (**Figure 1** **. G. H**).

### Relationship between CgA expression and SCCC patient survival based on IHC

Immunohistochemical assay showed that NSE was positive in 36 tumors (90.0%, 36/40), CgA in 27 tumors (67.5%, 27/40), and SYN in 25 tumors (62.5%, 25/40). Among, negative CgA expression (−/+) was observed in 13 SCCC specimens, with 8/40 (20.0%) labeled as (−) and 5/40 (12.5%) labeled as (+). In contrast, 27/40 (67.5%) SCCC specimens labeled with CgA expression levels of (++) or (+++) were recorded as positive (**Figure 2**). Univariate Cox analysis of the presence of neuroendocrine markers revealed that CgA was a poor prognostic factor (*P* = 0.003). Patient with CgA stained positive had poor survival (**Figure 3** **. A. B**), while SYN and NSE showed no impact on survival. Further analysis combined all 293 patients showed that CgA stained positive were significant predictors of poor survival in early-stage (FIGO I–IIa) patient specifically (*P*<0.05) (**Figure 3** **. C. D**), but not in late-stage (FIGO IIB–IV) (*P*>0.05) (**Figure 3** **. E. F**).

### Univariate and multivariate Cox analysis of all 293 SCCC patients to determine the poor prognostic factor

To identify factors prognostic for survival, we examined overall survival using Cox regression proportional hazard analyses. Univariate Cox regression analysis showed that FIGO stage (*P*<0.001), tumor mass size (*P*<0.001), lymph node metastasis (*P*<0.001), depth of stromal invasion (*P* = 0.022), and CgA stained positive (*P* = 0.034) were associated with prognostic of patients with SCCC. In contrast, age, tumor homology, lymph-vascular space invasion, NSE stained positive and SYN stained positive were not prognostic for survival (*P*>0.05) (Table 1). After adjusting for potential confounding factors, analyses using multivariate Cox regression model showed that FIGO stage of disease (HR, 3.08; 95% CI, 2.05–4.63; *P*<0.001), tumor mass size (HR, 2.37; 95% CI, 1.28–4.36; *P* = 0.006) and CgA stained positive (HR, 1.81; 95% CI, 1.12–2.91; *P* = 0.015) remained as significant independent prognostic factors for survival. Other factors, such as lymph node metastases, and depth of stromal invasion, were not significant independent prognostic factors for survival (**Table 1**).

## Discussion

Small cell carcinoma of the uterine cervix is an uncommon malignancy, accounting for 0.5–3.0% of all cervical cancers, is referred to by various names, such as small cell neuroendocrine tumor, small cell undifferentiated carcinoma, small cell carcinoma, argyrophilic cell carcinoma, and endocrine intermediate cell carcinoma. Similar to small cell lung cancer, cervical small cell carcinoma is difficult to manage and usually follows an aggressive clinical course, with death within a few years after diagnosis. The Gynecologic Oncology Group attempted to study small cell cervical carcinoma, but failed to recruit sufficient numbers of patients. As a result, treatment decisions have been based on these small single institution studies, and have extrapolated treatment approaches from the management of small cell cancer of the lung. The prognosis of SCCC is considered similar to that of small cell cancer of the lung, but long-term survival in SCCC patient has been reported [13]–[15]. To identify the clinical and pathologic factors prognostic of survival, and to determine optimal treatment strategies for patients with SCCC, 293 patients with SCCC reported in this study. To our knowledge, this is the largest study to date that analyzes the clinicopathologic and molecular markers associated survival outcomes.

Our data showed that early-stage disease is an independent prognostic factor. We observed a 3-year survival rate for all patients with SCCC of 34.5%, those with stage I–IIA disease had a 3-year survival of 48.4% compared with 13.0% for those with stage IIB–IV. Consistent with previous reports, the overall 3-year survivals ranged from approximately 50.0% for stage I patients, 40.0% for stage II patients, 25.0% for stage III patients, and under 10.0% for stage IV patients. In the current study, tumor size was also found to have a marginal significance in multivariate analysis (HR, 2.37; 95% CI, 1.28–4.36; *P* = 0.006). In fact, all these patients who were alive without evidence of disease at the time of last follow up were diagnosed at an early stage with lesions <2 cm. Similarly, Sheets et al. showed that patients with tumors <2 cm had longer progression-free survival than patients with >2 cm lesions [16]. With more patients, FIGO stage and tumor size were confirmed important in this rare cervical malignancy in our study and may ultimately prove to be an important independent prognostic predictor for survival.

Chromogranin A (CgA) is an acidic glycoprotein belonging to a family of regulated secretory proteins stored in the dense core granules of the adrenal medulla and of many other neuroendocrine cells and neurons. This protein is frequently used as a diagnostic and prognostic serum marker for a range of neuroendocrine tumors. In the present study, we observed that CgA was significantly increased in patients with SCCC, as previously reported in the literature. Moreover, our data showed that CgA stained positive is an independent prognostic factor of patients with SCCC (HR, 1.81; 95% CI, 1.12–2.91; *P* = 0.015). Interestingly, similar results were also observed in other human cancers, such as Straughn JM et al reported that CgA stained positive carried a significantly worse prognosis in patients with SCCC; tumors from the long-term survivors stained negative for CgA; patients with CgA -positive tumors were 21 times more likely to die (RR = 21.00) than patients negative for CgA (95% CI, 1.88–233.00). [17]. Whereas most published studies regarding CgA value on prostate cancer present encouraging results, Reis LO et al reported serum CgA as prognostic factor in high-risk prostate cancer [18]; CgA has been shown to correlate with disease severity, tumor volume, tumor burden, and overall prognosis [19]; Berruti A et al also reported tissue CgA expression, evaluated in prostate cancer needle biopsies at diagnosis, is an independent prognostic factor of survival in prostate cancer patients [20]. Recently, Malaguarnera M et al reported that CgA serum level as a marker of progression in hepatocellular carcinoma. patients with higher CgA level had poor survival and showed poor prognosis, compared to those with lower CgA level, the CgA is useful in monitoring progression of disease and may assist as a prognostic indicator [21]. A growing body of evidence suggests that CgA is more than a diagnostic marker for cancer patients. but evaluated as a significant prognostic marker in multiple human cancer. Recent findings implicating CgA could play important roles in tumor progression and response to therapy in cancer patients. Further study is necessary to assess the role of this protein and its fragments on the response to therapy and clarify the mechanisms on tumor outcome.

In conclusion, the results of the present study demonstrate that FIGO stage, tumor mass size and CgA stained may act as surrogate for factors prognostic of survival. Specifically, CgA stained positive should recommend to a novel marker useful in prognostic assessment for patients with SCCC. Although this study was retrospective in design and limited by the fact that the majority of patients were extracted from small case series making it difficult to validate the quality of information, it is the largest series reported to date. We hope that our experience contributes to the foundation of knowledge regarding this rare and aggressive tumor.

## Supporting Information

Table S1 **Review of literature published for small cell neuroendocrine carcinoma of the uterine cervix.**
(DOC)

Table S2 **The detail of immunohistochemistry methodology for Chromogranin A** (**CgA**)**.**
(DOC)

Table S3 **Univariate Analysis of Clinicopathological Factors Associated With the Chromogranin A Expression, Including 3-year OS and DFS Rates.**
(DOCX)
