# Supplementary material for: Chromogranin A (CgA) as Poor Prognostic Factor in Patients with Small Cell Carcinoma of the Cervix: Results of a Retrospective Study of 293 Patients
Source: PLoS One. 2012 Apr 17;7(4):e33674. doi: 10.1371/journal.pone.0033674 (PMC3328482; doi:10.1371/journal.pone.0033674)
Supplement: Table S1 — Review of literature published for small cell neuroendocrine carcinoma of the uterine cervix. (DOC) [file pone.0033674.s001.doc]

| **Table S1.** Review of literature published for small cell neuroendocrine carcinoma of the uterine cervix | | | | | | | | |
| --- | --- | --- | --- | --- | --- | --- | --- | --- |
| **Report authors** | **Number of cases** | **FIGO stage** | | |  | **Chromagranin A staining(CgA)** | | |
|  |  | **Ⅰ-ⅡA** | **ⅡB-Ⅳ** | **Survival outcome** |  | **Negative** | **Positive** | **Survival outcome** |
| From Cancer Center, Sun Yat-sen University, china | 47 | 36 | 7 | 11DoD(5-38M);24NED(2-119M) Vs. 4DoD(1-25M);3NED(20-63M) |  | 13 | 27 | 2DoD(16M and 20M);11NED(2-119M) Vs. 13DoD(5-38M);14NED(3-98M) |
| De-jie Liu et al. 2002 | 1 | / | 1 | DoD(1M) |  | / | 1 | DoD(1M) |
| Jun Tan et al. 2009 | 1 | 1 | / | DoD(16M) |  | / | 1 | DoD(16M) |
| Na Li et al. 2008 | 1 | 1 | / | NED(3M) |  | / | 1 | NED(3M) |
| Ling Tang et al. 2004 | 3 | / | 3 | 2DoD(10M and 13M);1AWD(5M) |  | / | / | / |
| Tian-Qi Guo et al. 1999 | 7 | 5 | 2 | 1DoD(5M);4NED(3-18M) Vs. 2DoD(4M and 7M) |  | / | / | / |
| Lin Xu et al. 2009 | 1 | 1 | / | DoD(7M) |  | / | 1 | DoD(7M) |
| Jun Hu et al. 2007 | 2 | 2 | / | 4NED(6M and 8M) |  | / | 2 | 4NED(6M and 8M) |
| Dong Wang et al. 2005 | 5 | 4 | 1 | 2DoD(9M and 17M);2NED(24-36M) Vs.DoD(10M) |  | 1 | 4 | DoD(10M)Vs. 2DoD(9M and 17M);2NED(24-36M) |
| Jun Wang et al. 2002 | 1 | 1 | / | DoD(12M) |  | / | 1 | DoD(12M) |
| Jian Wang et al. 1998 | 1 | / | 1 | DoD(2M) |  | 1 | / | DoD(2M) |
| Xiang-Li Wu et al. 2006 | 1 | 1 | / | NED(36M) |  | / | / | / |
| Tie-Gen Jiang et al. 2007 | 2 | 1 | 1 | NED(36M) Vs. DoD(10M) |  | / | 2 | 1DoD(10M);1NED(36M) |
| Hong-Lan Zhu et al. 2010 | 6 | / | 5 | 2DoD(3M and 17M);3AWD(1-8M) |  | / | 5 | 2DoD(13M and 17M);3AWD(1-8M) |
| Jian-Li Qu et al. 2002 | 1 | / | 1 | DoD(7M) |  | / | / | / |
| Chun-Mei Lu et al. 2003 | 6 | 3 | 3 | 1DoD(26M);1AWD(18M);1NED(19M) Vs.2DoD(11M and 12M);1NED(21M) |  | / | / | / |
| Guan-Jun Zhang et al. 2008 | 1 | 1 | / | NED(6M) |  | / | 1 | NED(6M) |
| Jia-Li Zhang et al. 2009 | 1 | / | 1 | DoD(2M) |  | / | 1 | DoD(2M) |
| Jing-Jing Yang et al. 2006 | 1 | 1 | / | AWD(13M) |  | / | / | / |
| Gui-Zhu Wu et al. 2009 | 6 | 3 | 3 | 1DoD(21M);2NED(2M and 16M)  Vs.DoD(17M);2NED(6M and 10M) |  |  | 6 | 2DoD(17M and 21M );4NED(2M and 16M) |
| Tie-Jun Zhou et al. 2007 | 4 | 2 | 2 | 1DoD(4M);1AWD(6M) Vs. 2DoD(1M and 5M) |  | 1 | 3 | DoD(4M) Vs. 2DoD(1M and 5M);1AWD(6M) |
| Yu Zhang et al. 2009 | 1 | 1 | / | DoD(13M) |  | / | / | / |
| Qiong-Tao Yang et al. 2002 | 2 | 1 | / | DoD(10M) |  | / | / | / |
| Chao Wang et al. 2008 | 9 | 9 | / | 8NED(7-44M) |  | 2 | 7 | 1NED(10M) Vs.7NED(7-44M) |
| Hong-Ying Yang et al. 2001 | 2 | 1 | 1 | NED(11M) Vs. DoD(10M) |  | / | 2 | 1NED(11M) Vs. 1DoD(10M) |
| Chen Zhou et al. 1998 | 13 | 10 | 3 | 3DoD(11-21M);7NED(2-118M) Vs.3NED(8-105M) |  | / | / | / |
| S.TSUNODA et al. 2005 | 11 | 4 | 7 | 1DoD(12M);2NED(44M and 144M) Vs.7DoD(4-16M) |  | 4 | 7 | 3DoD(4-13M);1NED(44M) Vs. 5DoD(8-16M);1NED(144M) |
| P.J.Hoskins et al. 1995 | 11 | 6 | 5 | 3DoD(8-20M);3NED(11-62M) Vs.4DoD(1-24M);1NED(10M) |  | / | / | / |
| John C Weed Jr et al. 2003 | 15 | 6 | 9 | 4DoD(11-44M);1NED(40M) Vs.9DoD(1-30M) |  | / | / | / |
| Nobuo Masumoto et al. 2003 | 10 | 7 | 3 | 3DoD(5-12M);2AWD(24M and 28M); 2NED(3M and 24M) Vs.3DoD(1-14M) |  | 1 | 7 | DoD(8M) Vs.3DoD(5-14M); 2AWD(24M and 28M);2NED(3M and 24M) |
| Akila N et al. 2004 | 21 | 17 | 4 | 11DoD(6-31M);6NED(25-209M)  Vs.4DoD(12-26M) |  | 4 | 17 | 4DoD(9-31M) Vs. 11DoD(6-27M);6NED(25-209M) |
| Lars-Christian Horn et al. 2006 | 9 | 7 | 1 | 4DoD(16-58M);3NED(50-151M) Vs.NED(102M) |  | / | / | / |
| S.Delaloge et al. 2000 | 10 | 6 | 4 | 4DoD(8-29M);2NED(13M and 53M) Vs.4DoD(10-19M) |  | 3 | 7 | 1DoD(29M);2NED(13M and 53M)  Vs.7DoD(10-28M) |
| Edgar Petru.C et al. 2005 | 10 | 2 | 8 | 1DoD(11M);1NED(14M) Vs.5DoD(6-31M); 1AWD(27M); 2NED(10M and 86M) |  | 6 | 4 | 5DoD(11-31M);1NED(14M) Vs.1DoD(6M) 1AWD(27M); 2NED(10M and 86M) |
| Min Jung Kim et al. 2008 | 13 | 9 | 1 | 6DoD(1-39M); 3NED(35-79M) Vs. DoD(19M) |  | 3 | 7 | 3NED(35-79M) Vs. 7DoD(1-39M) |
| Gabriela Mirei Ishida et al. 2004 | 10 | 6 | 4 | 1DoD(30M); 5NED(18-82M) Vs. 4DoD(13-28M) |  | 1 | 9 | 1DoD(28M) Vs. 4DoD(13-30M); 5NED(18-82M) |
| Tatsuki R.Kataoka et al. 2008 | 12 | 6 | 6 | 6NED(12-30M) Vs. 5DoD(6-18M);1NED(72M) |  | / | / | / |
| J.Michael Straughn Jr et al. 2001 | 16 | 11 | 5 | 7DoD(9-54M);1AWD(19M);3NED(16-264M) Vs.4DoD(6-43M);1NED(228M) |  | 8 | 8 | 4DoD(13-30M);1AWD(19M);3NED(60-264M); Vs.7DoD(6-40M);1NED(16M) |
| Micchitaka Ohwada et al. 2001 | 1 | 1 | / | NED(15M) |  | / | / | / |
| Fang-Kan Lim et al. 1999 | 1 | 1 | / | NED(54M) |  | 1 | / | NED(54M) |
| Tsuyoshi et al. 2008 | 1 | / | 1 | DoD(8M) |  | / | 1 | DoD(8M) |
| Surapan Khunamornpong et al. 2000 | 2 | 2 | / | 1DoD(12M);1AWD(9M) |  | / | 2 | 1DoD(12M);1AWD(9M) |
| Akihiko Watanabe et al. 2000 | 1 | / | 1 | DoD(11M) |  | / | 1 | DoD(11M) |
| Annna Reig Castillejo et al. 2010 | 1 | / | 1 | DoD(29M) |  | / | 1 | DoD(11M) |
| Woon-Kyong Chung et al. 2008 | 1 | / | / | / |  | 1 | / | AWD(48M) |
| Aylin Fidan et al. 2008 | 2 | 2 | / | 2NED(65M and 91M) |  | / | 2 | 2NED(65M and 91M) |
| Keith D. Balderston et al. 1998 | 1 | 1 | / | NED(60M) |  | / | 1 | NED(60M) |
| Akihiko Hashi et al. 1996 | 1 | / | 1 | DoD(8M) |  | / | 1 | DoD(8M) |
| Young B. Kim et al. 1996 | 1 | 1 | / | NED(92M) |  | / | 1 | NED(92M) |
| FIGO: Federation Internationale Gynecologica Obstetrica; M: months; DoD: died of disease; AWD: alive with disease; NED: no evidence of disease. | | | | | | | | |
