# Supplementary material for: Chromogranin A (CgA) as Poor Prognostic Factor in Patients with Small Cell Carcinoma of the Cervix: Results of a Retrospective Study of 293 Patients
Source: PLoS One. 2012 Apr 17;7(4):e33674. doi: 10.1371/journal.pone.0033674 (PMC3328482; doi:10.1371/journal.pone.0033674)
Supplement: Table S2 — The detail of immunohistochemistry methodology for Chromogranin A (CgA). (DOC) [file pone.0033674.s002.doc]

| **Table S3.** The detail of immunohistochemistry methodology for Chromogranin A (CgA) | | | | | | |  |
| --- | --- | --- | --- | --- | --- | --- | --- |
| **Reports authors** | **Antibody** | **clone** | **Source** | **Concentrations** | **Antigen** | **Scoring** |  |
|  | **against CGA** |  |  |  | **retrieval** | **method** |  |
| From Cancer Center,  Sun Yat-sen University, china | DAKO | polyclonal | rabbit | 1:300 | Yes | 1 |  |
| De-jie Liu et al. 2002 | DAKO | polyclonal | rabbit | 1:200 | Yes | 1 |  |
| Jun Tan et al. 2009 | DAKO | polyclonal | rabbit | / | / | 1 |  |
| Na Li et al. 2008 | / | / | / | / | / | / |  |
| Ling Tang et al. 2004 | DAKO | polyclonal | rabbit | 1:300 | Yes | 1 |  |
| Tian-Qi Guo et al. 1999 | / | / | / | / | / | / |  |
| Lin Xu et al. 2009 | BioGenex | polyclonal | rabbit | 1:200 | / | 1 |  |
| Jun Hu et al. 2007 [ | DAKO | polyclonal | rabbit | 1:300 | Yes | / |  |
| Dong Wang et al. 2005 | DAKO | polyclonal | rabbit | 1:250 | Yes | 1 |  |
| Jun Wang et al. 2002 | / | / | / | / | / | / |  |
| Jian Wang et al. 1998 | DAKO | polyclonal | rabbit | 1:350 | Yes | 1 |  |
| Xiang-Li Wu et al. 2006 | DAKO | polyclonal | rabbit | / | / | / |  |
| Tie-Gen Jiang et al. 2007 | BioGenex | polyclonal | rabbit | / | / | / |  |
| Hong-Lan Zhu et al. 2010 | DAKO | polyclonal | rabbit | 1:300 | Yes | 1 |  |
| Jian-Li Qu et al. 2002 | / | / | / | / | / | / |  |
| Chun-Mei Lu et al. 2003 | DAKO | polyclonal | rabbit | 1:150 | Yes | 1 |  |
| Guan-Jun Zhang et al. 2008 | DAKO | polyclonal | rabbit | 1:300 | / | 1 |  |
| Jia-Li Zhang et al. 2009 | DAKO | polyclonal | rabbit | 1:250 | Yes | 1 |  |
| Jing-Jing Yang et al. 2006 | / | / | / | / | / | / |  |
| Gui-Zhu Wu et al. 2009 | DAKO | polyclonal | rabbit | 1:300 | / | / |  |
| Tie-Jun Zhou et al. 2007 | DAKO | polyclonal | rabbit | 1:300 | Yes | 1 |  |
| Yu Zhang et al. 2009 | DAKO | polyclonal | rabbit | 1:250 | / | 1 |  |
| Qiong-Tao Yang et al. 2002 | / | / | / | / | / | / |  |
| Chao Wang et al. 2008 | DAKO | polyclonal | rabbit | 1:250 | Yes | 1 |  |
| Hong-Ying Yang et al. 2001 | DAKO | polyclonal | rabbit | 1:300 | Yes | 1 |  |
| Chen Zhou et al. 1998 | DAKO | polyclonal | rabbit | 1:150 | / | / |  |
| S.TSUNODA et al. 2005 | DAKO | polyclonal | rabbit | 1:200 | Yes | 1 |  |
| P.J.Hoskins et al. 1995 | BioGenex | polyclonal | rabbit | 1:150 | Yes | 1 |  |
| John C Weed Jr et al. 2003 | / | / | / | / | / | / |  |
| Nobuo Masumoto et al. 2003 | BioGenex | polyclonal | rabbit | 1:150 | Yes | 1 |  |
| Akila N et al. 2004 |  |  |  |  |  |  |  |
| Lars-Christian Horn et al. 2006 | BioGenex | polyclonal | rabbit | 1:200 | Yes | 1 |  |
| S.Delaloge et al. 2000 | / | / | / | / | / | / |  |
| Edgar Petru.C et al. 2005 | / | / | / | / | / | / |  |
| Min Jung Kim et al. 2008 | / | / | / | / | / | / |  |
| Gabriela Mirei Ishida et al. 2004 | Immunotech | LK2H10 | rabbit | / | Yes | 1 |  |
| Tatsuki R.Kataoka et al. 2008 | / | / | / | / | / | / |  |
| J.Michael Straughn Jr et al. 2001 | BioGenex | polyclonal | rabbit | 1:150 | / | / |  |
| Micchitaka Ohwada et al. 2001 | / | / | / | / | / | / |  |
| Fang-Kan Lim et al. 1999 | DAKO | polyclonal | rabbit | 1:250 | Yes | 1 |  |
| Tsuyoshi et al. 2008 | / | / | / | / | / | / |  |
| Surapan Khunamornpong et al. 2000 | / | / | / | / | / | / |  |
| Akihiko Watanabe et al. 2000 | Nichirei | polyclonal | rabbit | 1:100 | / | 1 |  |
| Annna Reig Castillejo et al. 2010 | / | / | / | / | / | / |  |
| Woon-Kyong Chung et al. 2008 | DAKO | polyclonal | rabbit | / | / | / |  |
| Aylin Fidan et al. 2008 | BioGenex | polyclonal | rabbit | 1:150 | / | 1 |  |
| Keith D. Balderston et al. 1998 | DAKO | polyclonal | rabbit | 1:300 | Yes | 1 |  |
| Akihiko Hashi et al. 1996 | / | / | / | / | / | / |  |
| Young B. Kim et al. 1996 | DAKO | polyclonal | rabbit | 1:200 | / | / |  |
| **/: unknow; 1: immunoreactivity was described by the percentage of positive tumor cells and by the staining intensity** | | | | | | | |
