# Supplementary material for: Chromogranin A (CgA) as Poor Prognostic Factor in Patients with Small Cell Carcinoma of the Cervix: Results of a Retrospective Study of 293 Patients
Source: PLoS One. 2012 Apr 17;7(4):e33674. doi: 10.1371/journal.pone.0033674 (PMC3328482; doi:10.1371/journal.pone.0033674)
Supplement: Table S3 — Univariate Analysis of Clinicopathological Factors Associated With the Chromogranin A Expression, Including 3-year OS and DFS Rates. (DOCX) [file pone.0033674.s003.docx]

| **Table S2.** Univariate Analysis of Clinicopathological Factors Associated With the Chromogranin A Expression , Including 3-year OS and DFS Rates | | | | | | | | | |
| --- | --- | --- | --- | --- | --- | --- | --- | --- | --- |
| **Variables** | **The number of** | **Number(%)** | **Chromogranin A** | | | **3-year DFS(%)** | **P Value** | **3-year OS(%)** | **P Value** |
|  | **missing patients** |  | **(-)** | **(+)** | **P Value** |  |  |  |  |
| **Age(years)** | 0 |  |  |  |  |  |  |  |  |
| <40 |  | 141(48.1) | 18 | 60 |  | 34.2 |  | 39.2 |  |
| ≥40 |  | 152(51.9) | 32 | 78 | 0.358 | 28.0 | 0.553 | 30.9 | 0.270 |
| **FIGO stage** |  |  |  |  |  |  |  |  |  |
| Ⅰ-Ⅱa | 9 | 186(65.5) | 31 | 97 |  | 43.6 |  | 48.4 |  |
| Ⅱb-Ⅳ |  | 98(34.5) | 19 | 41 | 0.183 | 12.6 | **<0.001** | 13.0 | **<0.001** |
| **Lymph node metastasis** | 108 |  |  |  |  |  |  |  |  |
| Positive |  | 87(47.0) | 9 | 47 |  | 14.4 |  | 23.4 |  |
| Negative |  | 98(53.0) | 15 | 54 | 0.424 | 52.6 | **<0.001** | 54.1 | **<0.001** |
| **Tumor mass size** | 136 |  |  |  |  |  |  |  |  |
| <4cm |  | 72(45.9) | 10 | 49 |  | 47.3 |  | 58.4 |  |
| ≥4cm |  | 85(54.1) | 15 | 41 | 0.201 | 18.7 | **<0.001** | 20.1 | **<0.001** |
| **Lymph-vascular space invasion** | 173 |  |  |  |  |  |  |  |  |
| Positive |  | 65(54.2) | 12 | 40 |  | 26.6 |  | 33.5 |  |
| Negative |  | 55(46.8) | 12 | 33 | 0.683 | 39.3 | 0.094 | 46.7 | 0.214 |
| **Tumor homology** | 88 |  |  |  |  |  |  |  |  |
| Pure |  | 147(71.7) | 23 | 77 |  | 35.9 |  | 42.8 |  |
| Mixed |  | 58(28.3) | 5 | 29 | 0.304 | 32.0 | 0.214 | 35.6 | 0.499 |
| **Depth of stromal invasion** | 199 |  |  |  |  |  |  |  |  |
| <2/3 |  | 40(42.6) | 5 | 26 |  | 46.1 |  | 59.9 |  |
| ≥2/3 |  | 54(57.4) | 8 | 37 | 0.566 | 26.2 | **0.013** | 34.9 | **0.018** |
| **Therapy** | 33 |  |  |  |  |  |  |  |  |
| RH / CT / RT/RH+RT /CT+RT/other |  | 93 | 18 | 51 |  | 17.6 |  | 19.7 |  |
| RH+CT/RH+CT+RT |  | 167 | 29 | 82 | 0.569 | 41.9 | **0.012** | 43.6 | **0.008** |
| **Neurone-specific enolase** | 131 |  |  |  |  |  |  |  |  |
| Positive |  | 137(84.6) | 32 | 93 |  | 27.3 |  | 30.8 |  |
| Negative |  | 25(16.4) | 7 | 15 | 0.189 | 42.4 | 0.521 | 53.8 | 0.426 |
| **Synaptophysin** | 132 |  |  |  |  |  |  |  |  |
| Positive |  | 131(81.4) | 23 | 103 |  | 28.1 |  | 31.0 |  |
| Negative |  | 30(18.6) | 16 | 14 | **<0.001** | 47.6 | 0.132 | 50.1 | 0.088 |
| **cytokeratin** | 130 |  |  |  |  |  |  |  |  |
| Positive |  | 118(72.4) | 18 | 70 |  | 27.3 |  | 29.4 |  |
| Negative |  | 45(27.6) | 6 | 24 | 0.592 | 30.6 | 0.535 | 32.1 | 0.612 |
| **CD56** | 167 |  |  |  |  |  |  |  |  |
| Positive |  | 93(73.8) | 11 | 52 |  | 21.6 |  | 23.2 |  |
| Negative |  | 33(26.2) | 4 | 12 | 0.356 | 40.3 | 0.114 | 42.3 | 0.148 |
| **CD45** | 169 |  |  |  |  |  |  |  |  |
| Positive |  | 78(62.9) | 14 | 24 |  | 22.4 |  | 23.9 |  |
| Negative |  | 46(37.1) | 9 | 18 | 0.490 | 39.1 | 0.234 | 41.6 | 0.189 |
| **Ki67** | 217 |  |  |  |  |  |  |  |  |
| Positive |  | 65(85.5) | 7 | 28 |  | 22.5 |  | 24.5 |  |
| Negative |  | 11(14.5) | 2 | 5 | 0.472 | 43.8 | 0.071 | 45.7 | 0.077 |
| **Chromogranin A** | 105 |  |  |  |  |  |  |  |  |
| Positive |  | 138(73.4) | / | / |  | 27.5 |  | 30.0 |  |
| Negative |  | 50(26.6) | / | / | / | 40.3 | 0.052 | 46.8 | 0.053 |
| (+): Patients with Chromogranin A stained positive; (-): Patients with Chromogranin A stained negative; **Bold** indicates signiﬁcant values. | | | | | | | |  |  |
| RH: radical hysterectomy; CT: chemotherapy; RT:radiotherapy; | | | | | | | | | |
